# Reasons for non-participation of children and adolescents in a large-scale school-based mental health project

**DOI:** 10.3389/fpubh.2023.1294862

**Published:** 2024-01-08

**Authors:** Sabrina Baldofski, Sarah-Lena Klemm, Elisabeth Kohls, Sophia M. E. Mueller, Stephanie Bauer, Katja Becker, Silke Diestelkamp, Heike Eschenbeck, Alisa Hiery, Michael Kaess, Julian Koenig, Laya Lehner, Markus Moessner, Rainer Thomasius, Christine Rummel-Kluge

**Affiliations:** ^1^Department of Psychiatry and Psychotherapy, Medical Faculty, University of Leipzig, Leipzig, Germany; ^2^Department of Psychiatry and Psychotherapy, University of Leipzig Medical Center, Leipzig, Germany; ^3^Center for Psychotherapy Research, University Hospital Heidelberg, Heidelberg, Germany; ^4^Department of Child and Adolescent Psychiatry, Psychosomatics and Psychotherapy, Faculty of Medicine, Philipps-University and University Hospital Marburg, Marburg, Germany; ^5^Center for Mind, Brain and Behavior (CMBB), University of Marburg and Justus Liebig University Giessen, Giessen, Germany; ^6^German Center for Addiction Research in Childhood and Adolescence, University Medical Center Hamburg-Eppendorf, Hamburg, Germany; ^7^Department of Educational Psychology and Health Psychology, University of Education Schwäbisch Gmünd, Schwäbisch Gmünd, Germany; ^8^University Hospital of Child and Adolescent Psychiatry and Psychotherapy, University of Bern, Bern, Switzerland; ^9^Department of Child and Adolescent Psychiatry, Center for Psychosocial Medicine, University Hospital Heidelberg, Heidelberg, Germany; ^10^Department of Child and Adolescent Psychiatry, Psychosomatics and Psychotherapy, Faculty of Medicine and University Hospital Cologne, University of Cologne, Cologne, Germany

**Keywords:** mental health, children, adolescence, non-participation, school-based study, online intervention

## Abstract

**Background:**

Non-participation in mental health studies is an under-explored but very important topic. Investigating reasons for non-participation holds promise for the planning of future study designs and recruitment strategies. This study aimed at investigating reasons for children and adolescents (C&A) not participating in a school-based mental health research project.

**Methods:**

Data collection took place within the school-based recruitment of a large-scale multi-site project (“ProHEAD—Promoting Help-seeking using E-technology for Adolescents”) in Germany. Participants were *N* = 534 C&A aged ≥ 12 years attending secondary schools. The present cross-sectional study analyzed anonymous survey data of C&A who themselves or whose parents, respectively, did not provide written consent to participate in the mental health research project. The questionnaire consisted of 14 items covering potential reasons for non-participation, and four free text fields. Besides descriptive statistics, free text field answers were analyzed using qualitative content analysis.

**Results:**

Students indicated an average of *M* = 2.94 (*SD* = 1.75) reasons for their non-participation in the project. In the descriptive analysis of indicated items, the three most frequently reported reasons for non-participation included students reporting to not be concerned by the topic “mental health” (*n* = 290, 54.3%), not having returned the consent form to the teacher (*n* = 175, 32.8%), and not having time for participation (*n* = 149, 27.9%). In the qualitative content analysis, the most frequently assigned categories were organizational reasons (*n* = 216, 57.1%), general disinterest in study participation (*n* = 139, 36.8%), and personal attitudes toward the topic “mental health” (*n* = 84, 22.2%), such as not being concerned with the topic “mental health” (*n* = 23, 6.1%) or being too concerned with the topic “mental health” (*n* = 16, 4.2%).

**Conclusion:**

The study provides unique insights into reasons for C&A and their caregivers not participating in a large federally funded mental health research project. The results suggest that in order to increase participation rates, stigma should be reduced, parents as well as teachers should be involved where possible, and the use of incentives might be helpful. The study highlights the importance of assessing reasons for non-participation, especially in online intervention studies on mental health.

## Introduction

Child and adolescent mental health has been worrisome to medical experts and researchers for the past decades (1), deteriorating especially in the past few years during the COVID-19 pandemic (2). With a worldwide prevalence of 10–20% (1–3), mental disorders such as depression, anxiety disorders, and attention deficit hyperactivity disorder (ADHD) are among the most common disorders in the age group of 14–25 year olds (4). Since the onset of a mental disorder at an early stage in life is a strong predictor for mental disorders in later life (5), the need for feasible and effective mental health support (i.e., mental health promotion, prevention, and treatment) for children and adolescents (C&A) is evident. However, access to such services is currently still extremely limited, with only a quarter of all C&A affected by mental disorders receiving professional treatment (6).

Considering the high individual and societal costs and burden of mental health issues, the urgency for high quality research on C&A is strongly supported. High participation rates in studies on mental health are needed to maximize generalizability of study results and thus, gain meaningful insights on C&A's mental health status and on the feasibility and effectiveness of interventions aiming at the prevention or treatment of mental health problems. To date, non-participation in studies on mental health is a hardly explored topic. Low participation rates may reduce statistical power and increase the risk for selection bias. Therefore, it is of importance to gain knowledge on attitudes toward study participation and potential barriers: Why do some individuals voluntarily engage and participate in studies and mental health interventions, while others do not? While every successful study process (and especially the recruitment of participants) relies on the participation of volunteers, finding out more about the non-participants and their reasons for choosing not participating is a highly relevant research topic. Non-participation has been extensively researched in the field of epidemiological studies (7), however, studies investigating non-participation in mental health research projects, especially focusing on C&A, are scarce. Recruitment for (online) mental health research is known to present various difficulties, as individuals may be hesitant to participate due to stigma or privacy concerns, often resulting in low participation rates (8). However, sufficient participation rates seem to be especially important in online intervention studies on mental health, as these interventions may play a key role in reaching different target groups and increasing help-seeking behavior by providing low-threshold access to mental health support.

The present study was conducted nested within a large-scale school-based mental health project in Germany, the “ProHEAD” project (Promoting Help-seeking using E-technology for Adolescents). The main goal of ProHEAD is to assess new access pathways for prevalent mental health problems in C&A. Within the ProHEAD consortium, C&A were allocated to one of five RCTs based on an initial screening questionnaire (9). The RCTs focused on an improvement of help-seeking in participants with clinically relevant symptoms (10), prevention in participants at risk for mental disorders (11–13), and mental health promotion in participants without mental health problems (14). The present study aimed at understanding and exploring reasons for C&A not participating in the ProHEAD project. To this end, data from an anonymous questionnaire were analyzed using quantitative and qualitative methods.

## Methods

### Participants and procedure

Data collection for the present study took place within the recruitment of the ProHEAD project. The ProHEAD project (Promoting Help-seeking using E-technology for Adolescents) is a large-scale multi-site consortium in Germany (2017–2023) (10). The recruitment was school-based and took place in five urban areas geographically distributed across Germany (Hamburg, Heidelberg, Leipzig, Marburg, and Schwäbisch Gmünd). The aim of ProHEAD is to assess mental health problems in C&A longitudinally and to examine the effectiveness of different online interventions for the prevention and treatment of mental health problems as well as mental health promotion. After completing a school-based computerized baseline screening questionnaire, participants were allocated to one of five RCTs and received an invitation to participate in an online intervention. The intervention period was followed by school-based computerized 1- and 2-year follow-up assessments. While follow-up assessments within the ProHEAD project are still ongoing, baseline screening assessments of the project were completed, with a sample size of *N* = 9,509 C&A having completed the screening questionnaire across all study centers.

Participants in the present study were students aged ≥12 years attending secondary schools (school grades 6–13) in one of the recruiting centers of the ProHEAD consortium (Leipzig). The recruitment within the ProHEAD project took place in a school context, where researchers held classroom presentations for the students in their respective class to introduce the research project. Each student received information material and consent forms—one consent form for the student themselves and, in the case of students under the legal age of 18, one consent form to be handed to their parents and to be signed by one parent or legal guardian.

The completed consent forms were afterwards collected by the respective teacher and personally collected at the school by the recruitment team to protect the sensitive data of participants [for details see Kaess and Bauer (10), Baldofski et al. (11), Diestelkamp et al. (13), Eschenbeck et al. (14), and Kaess et al. (9)]. Within the school-based recruitment of the ProHEAD project in Leipzig, students who did not provide written informed consent (i.e., consent from the participants themselves and/or parental consent for underage participants) to participate in the ProHEAD project were asked to fill in an anonymous one-page paper-and-pencil questionnaire. In total, students from *n* = 8 different schools participated in the anonymous survey. Before completing the questionnaire, students were informed about the anonymity of the questionnaire and the voluntary nature of their participation. The completed questionnaires were afterwards sealed by the school staff and sent back to the recruitment center.

In the *n* = 8 participating schools in Leipzig, a total of *N* = 2,535 students were informed about the study. Of these, *n* = 828 (32.7%) provided written informed consent to participate in the ProHEAD project. Of the remaining *n* = 1,707 (67.3%) who did not provide consent, *n* = 534 (31.3%) students completed the anonymous questionnaire on non-participation.

Data were collected between December, 2018 and March, 2021. Ethical approval for the anonymous survey was granted by the Ethics Committee of the Medical Faculty, University of Leipzig, on November 28, 2018 (file reference: 181/18-lk).

### Measures

#### Non-participation questionnaire (NPQ)

For the purpose of this study, a questionnaire assessing potential reasons for non-participation was developed by researchers of the recruiting ProHEAD center (Leipzig). The items were developed in a consensus process including senior psychiatrists, clinical psychologists and other mental health professionals, C&A, teachers, and school social workers. In addition, a literature review on school-based recruitment and non-participation in research projects was performed.

The resulting Non-Participation Questionnaire (NPQ) is a short (one-page) questionnaire including 14 items and four open text fields. The items include statements on potential reasons for non-participation in the mental health research project, covering different domains of potential reasons such as organizational reasons, reasons related to the parents' consent, and personal reasons, including attitudes toward mental health (for details on items see Table 1). Each item constitutes a different reason for non-participation, and each reason could be indicated by checking a box next to each item. Students were informed in the instruction that they could indicate multiple reasons. Therefore, it was possible to indicate a maximum of 14 different reasons in total. In addition to these items, four open text fields were provided to give participants the option to further explain specific reasons for non-participation. Specifically, further explanations or specific reasons could be given for the items “No time to participate,” “Feeling uncomfortable with the topic mental health,” and “Consent form was not returned to teacher.” Another open text field was included at the end of the questionnaire, where any other reasons or further explanations could be given.

**Table 1:** Descriptive analysis of reasons for non-participation (*N* = 534).

**Variable, *n* (%)^a^**	**Students in secondary schools (*N* = 534)**
Topic (mental health) does not concern me	290 (54.3)
Consent form was not returned to teacher	175 (32.8)
No time to participate	149 (27.9)
Concerns (from self or parents) regarding data (personal information) being stored	141 (26.4)
No interest in the topic (mental health)	132 (24.7)
Friends do not participate either	119 (22.3)
Parents do not want participation	104 (19.5)
No financial compensation is offered	99 (18.5)
Consent form is too long and complicated	95 (17.8)
Lack of understanding the project's scope	92 (17.2)
Fear that people will think I am crazy or ill	52 (9.7)
Feeling uncomfortable with the topic (mental health)	50 (9.4)
Parents did not understand what the project is about	37 (6.9)
Information on the project was not received	37 (6.9)

a Multiple answers were possible. Calculation of % from valid cases.

### Statistical analysis

First, a quantitative descriptive analysis was performed to examine the number of reasons for non-participation given in total (i.e., number of items indicated) and the frequency of each reason. A chi square test was conducted to evaluate the association between students that had indicated that they were not concerned by the topic mental health and not handing back the IC to the teacher.

Second, a qualitative analysis was performed. To this end, the qualitative data of the four open text field answers were coded using MAXQDA qualitative software (version 2022.0.0). The qualitative analysis was based on Mayring's summarizing content analysis (15). Following this approach, a coding dictionary was developed to analyze the open text field answers. Allowing the themes to emerge from the raw data, the codes represented themes given as reasons for non-participation. To represent every open text statement, the coding was conducted with the aim to develop as few coding categories as possible, but as many as necessary. If a participant gave several open text statements which fell into the same coding category, the respective coding category was only assigned once to the respective participant to avoid distortion of frequencies.

Using the final coding manual, all open text field data were coded by one author. In addition, inter-rater reliability was computed to ensure the validity of the coding. To this end, a subset of 25% of all data was randomly selected and coded by a second researcher who was unfamiliar with the study. Ratings were then compared (16) and resulted in an estimated inter-rater reliability of κ = 0.90, which was based on a mean-rating (*k* = 2), absolute-agreement, 2-way mixed-effects model. This estimation is indicative of an excellent reliability (17) and thus, suggests a high validity of the coding manual. After coding, frequencies of all coding categories were descriptively analyzed.

## Results

### Study sample

In total, *N* = 534 students completed the questionnaire. Of these, a subsample of *n* = 378 (70.8%) students filled in one or more open text fields with *n* = 470 statements in total.

### Quantitative analysis

Regarding the number of reasons for non-participation given in total, students indicated an average of *M* = 2.94 (*SD* = 1.75; range 0–14) reasons. In total, *n* = 461 (86.3%) students indicated up to four reasons, while *n* = 31 (5.8%) did not indicate any reason in the multiple-choice items, but filled in one or more open text fields.

The frequency of each reason for non-participation is detailed in Table 1. The three most frequently indicated reasons included students reporting to not be concerned by the topic “mental health” (*n* = 290, 54.3%), not having returned the consent form to the teacher (*n* = 175, 32.8%), and not having time for participation (*n* = 149, 27.9%). Further reasons included concerns regarding data privacy (i.e., student or parents not wanting personal information being stored; *n* = 141, 26.4%), no interest in the topic “mental health” (*n* = 132, 24.7%), and not participating because one's friends also did not participate (*n* = 119, 22.3%). Of *n* = 290 students who indicated that the topic mental health does not concern them, *n* = 95 (17.7%) also indicated that they did not return the IC to the teacher, while *n* = 195 (36.5) indicated that the topic mental health does not concern them, but did not reported that they did not hand the IC back to the teacher. A chi square test showed that there was no significant association between not being concerned by the topic mental health and not handing in the IC back to the teacher [*X*^2^_(1.534)_ = 0.00, *p* = 0.994].

### Qualitative content analysis

The final coding manual consisted of 24 categories, which included three main reasons for non-participation: general disinterest in study participation, as well as critique and doubts of study goals and procedures (six categories); organizational reasons, such as reasons regarding the consent form, lack of time, or inclusion criteria (minimum age; 10 categories); and personal attitudes toward the topic “mental health” and individual reasons (seven categories); as well as one category of non-assignable answers (see Table 2 for an overview of categories and examples).

**Table 2:** Qualitative content analysis (Categories of reasons for non-participation (free text field answers) in the final coding manual).

**Categories and sub-categories**	**Example of free text field answers**
**General disinterest in study participation, as well as critique and doubts of study goals and procedures**	
No interest	“I don't want to participate”
Parents do not agree to let the student participate	“My parents don't want me to participate”
Friends do not participate	“I don't want to do it alone without my friends”
Concerns about data privacy	“Not anonymous, information could be traced back to us”
Expectation of financial compensation for participating	“I would have really liked to receive money”
Doubts regarding the effectiveness of online programs	“I spare myself from so called ‘help' through a program”
**Organizational reasons such as reasons regarding the consent form, lack of time, or inclusion criteria (minimum age)**	
Information regarding the project was not received	“I did not get the letter”
Consent form was forgotten	“I forgot the letter”
Other organizational problems regarding the consent form	“My mother signed the form too late”
Not wanting to miss school in order to participate	“I don't want to miss class”
Not wanting to invest free time in order to participate	“I have a lot of other hobbies and thus no time. Sorry.”
Information regarding the project was not understood	“I don't know what the program was about”
More information regarding the project was needed	“I did not get enough information (on risks)”
Participation not possible due to absence	“I was sick”
Minimum age of 12 not reached	“I'm not 12 yet”
Shared e-mail address	“I don't have my own e-mail address”
**Personal attitudes toward the topic “mental health” and individual reasons**	
Not concerned with the topic “mental health”	“I didn't participate because it does not affect me”
Too concerned with the topic “mental health” to participate	“Because I'm already dealing with it”
Already receiving psychological treatment	“I am already in therapy”
Preoccupied with personal and family issues	“I am busy with the household and problems within the family”
Belief that private matters should not be talked about	“I don't think it's good to talk to people that I don't know”
Talking about mental health is uncomfortable	“I don't like to talk about it”
Stigma or negative attitudes toward mental health and professional help	“I'm not a guinea pig”
**Non-assignable**	
Non-assignable	“It is what it is”

Regarding frequencies of the main reasons for non-participation, the most frequently assigned category was “organizational reasons” (indicated by *n* = 216, 57.1% students), with the most frequent sub-categories being “not wanting to miss school in order to participate” (*n* = 55, 14.6%) and “having forgotten the consent form” (i.e., the student did not return the consent form to their teacher; *n* = 52, 13.8%; see Table 3). The second most frequently assigned category was “general disinterest in study participation” (*n* = 139, 36.8%), with the most frequent sub-category “being not interested in participating” (*n* = 97, 25.7%). Finally, the category of “personal attitudes toward the topic mental health” was assigned to statements of *n* = 84 (22.2%) students, with the most frequent sub-categories “being not concerned with the topic mental health” (*n* = 23, 6.1%) and “being too concerned with the topic mental health” (*n* = 16, 4.2%), respectively.

**Table 3:** Frequencies of reasons for non-participation (free text field answers of *N* = 378 students).

**Categories and sub-categories, *n* (%)**	**Students in secondary schools (*N* = 378)**
**General disinterest in study participation, as well as critique and doubts of study goals and procedures**	139 (36.8)
No interest	97 (25.7)
Parents do not agree to let the student participate	13 (3.4)
Friends do not participate	3 (0.8)
Concerns about data privacy	10 (2.7)
Expectation of financial compensation for participating	3 (0.8)
Doubts regarding the effectiveness of online programs	13 (3.4)
**Organizational reasons such as reasons regarding the consent form, lack of time, or inclusion criteria (minimum age)**	216 (57.1)
Information regarding the project was not received	11 (2.9)
Consent form was forgotten	52 (13.8)
Other organizational problems regarding the consent form	17 (4.5)
Not wanting to miss school in order to participate	55 (14.6)
Not wanting to invest free time in order to participate	28 (7.4)
Information regarding the project was not understood	4 (1.1)
More information regarding the project was needed	6 (1.6)
Participation not possible due to absence	18 (4.8)
Minimum age of 12 not reached	22 (5.8)
Shared e-mail address	3 (0.8)
**Personal attitudes toward the topic “mental health” and individual reasons**	84 (22.2)
Not concerned with the topic “mental health”	23 (6.1)
Too concerned with the topic “mental health” to participate	16 (4.2)
Already receiving psychological treatment	12 (3.2)
Preoccupied with personal and family issues	9 (2.4)
Belief that private matters should not be talked about	13 (3.4)
Talking about mental health is uncomfortable	7 (1.9)
Stigma or negative attitudes toward mental health and professional help	4 (1.1)
**Non-assignable**	31 (8.2)
Non-assignable	31 (8.2)

## Discussion

This study aimed to gain a better understanding of reasons for C&A not participating in a large school-based mental health project, using data from an anonymous questionnaire specifically developed for the purpose of this study. As shown in the descriptive analysis of questionnaire items, students gave an average three reasons for non-participation, with the main reason of not being concerned by the topic “mental health,” and other reasons including not having returned the consent form to the teacher and not having time for participation. A qualitative content analysis of the open text field answers identified organizational reasons, a general disinterest in study participation, and personal attitudes toward the topic “mental health” as the most frequent reasons for non-participation.

The study results give a detailed insight into the barriers to participating in a school-based intervention study on mental health in children and young adults. While 54.3% of students indicated to not be concerned by the topic “mental health,” 4.2% of all students having answered the open text fields reported that they were too concerned with the topic. Examples for the latter category included reports of being diagnosed with a mental disorder, or being preoccupied with own mental health problems, e.g., dealing with grief. Further, 3.2% indicated in the open text fields that they were already receiving psychological treatment and thus did not want to participate. For an overall understanding of the results of this study, it is important to highlight that mental health in general is still a stigmatizing topic for young people, and that stigma presents a barrier to seeking and accessing professional help when needed (18–21). The result on not being concerned by the topic “mental health” may reflect this underlying stigma. In turn, the result on being too concerned by the topic may reflect an increased burden by mental health problems, which might be present in students themselves, their parents, family, or friends. Being too concerned could also be interpreted as the fear of a personal mental health deterioration, as a consequence of a participation in ProHEAD, as some students described in the free text fields.

Stigmatizing attitudes toward mental health were also represented in the findings of 9.7% of students indicating a fear that people would think they are crazy or ill when participating in the study, and 9.4% reporting to feel uncomfortable with the topic. Interestingly there was no association between the proportion of students indicating that mental health does not concern them and the proportion of students that did not hand the IC back to the teacher. During the recruitment process and the presentation of the research project to the students, it was emphasized that the project and the online interventions were targeted toward all students [including students currently not affected by mental health problems (14)], regardless of their previous experiences with or knowledge about the topic. However, the fact that over half of all participants indicated to not be concerned by the topic “mental health” suggests that a substantial proportion of C&A do not perceive mental health as a normal part of their everyday life. Further, a general lack of interest in the project and the topic, as expressed by 24.7 and 25.7% in the indicated items and the open text fields, respectively, might be explained by attitudes specific to this age group (22).

Moreover, different organizational reasons were reported as reasons for non-participation, the largest of them being reasons regarding the consent form. Specifically, 32.8% of students indicated in the questionnaire items to not have returned the consent form to the teacher, while in the open text fields, 18.3% reported to have forgotten the consent form or to have had other organizational problems regarding the consent form. To facilitate the consent process, the paper consent forms were turned into digital forms in the course of the study and during the COVID-19-pandemic, thus reducing the risk of participant loss through student disorganization. Further, being younger than 12 years and thus not meeting inclusion criteria was reported by 5.8% in the open text fields as another reason for non-participation. Due to school-related organizational reasons, some students in grade six had not yet reached the age of twelve, but took part in the informative classroom presentation. Some of them expressed their disappointment for not being able to take part in the study. To prevent such frustration in future recruitment, it would be advisable to only present the study to possibly eligible participants or to adapt inclusion criteria (i.e., students in grade 6 rather than students of a specific age). Other reasons for non-participation also arose from school-related organizational reasons. Specifically, 2.9% stated in the open text fields that they had not received any information regarding the research project, while 4.8% were absent on the day of the classroom presentation.

Time, or the lack thereof, seems to be another important reason for non-participation and was indicated by 27.9% of the students in the questionnaire items. In the open text fields, preoccupation with school, extracurricular activities, and hobbies were common reasons reported by the students. It is of note that indicating a lack of time, rather than other possible reasons for non-participation presented in the questionnaire, may also reflect a social desirability effect. In total, 7.4% of students expressed their unwillingness to invest free time in order to participate in the open text fields. In contrast to this finding, a significant proportion of 14.6% of students expressed their unwillingness to miss school lessons in order to participate in the research project. The computerized baseline screening questionnaires for the project were school-based and took place for all participants of a respective class during regular school hours. While developing the study design and recruitment process, it was anticipated that participating in the context of one's own class and during school hours might maximize participation rates. Further, it was initially planned that baseline screenings would take place during free periods, so that no student would miss relevant school lessons, but due to school-related organizational reasons this procedure could not always be implemented. For future studies, planning two potential time slots (one during class and one during a free period) for completion of questionnaires could be helpful to increase participation rates. Moreover, offering to complete the questionnaires online at home might be helpful, which was also done in the course of the ProHEAD project due to pandemic-related restrictions preventing the research team to visit schools in person.

During the development period of the Non-Participation Questionnaire, it was assumed—based on relevant literature (17)—that a student's peer group would have an influence on their choice to participate in the research project. This assumption could be confirmed in the results, with 22.3% of students indicating in the questionnaire items that they choose not participating because their friends also did not participate. In addition to the role of peers, the role of parental support and approval is also essential, especially considering the fact that most participants in the research project were underage and thus, written consent of one of their parents was required for participation. Specifically, 19.5% of students indicated in the questionnaire items that their parents did not want them to participate. This result might also be linked to the abovementioned findings on a significant proportion of students who had not returned the consent form to their teacher. During the recruitment for the research project, letters and flyers for the parents were included when handing out informational materials and informed consent forms to the students during the classroom presentations in order to also inform parents about the project. For future studies, parents should be involved in the recruitment process where possible, e.g., information on a research project could be given during parents' class meetings.

Strengths of this study include the large sample size (representing 31.3% of all students in the participating schools who did not provide informed consent for the ProHEAD project) as well as the anonymous nature of data collection, increasing the probability of truthful answers. However, due to the face-to-face nature of the recruitment in the class context, it could be argued that the present study was not totally anonymous and students might have feared negative conclusions regarding their person when answering the Non-Participation Questionnaire.

Another strength is the mixed-methods approach including a descriptive quantitative analysis and a qualitative content analysis. A limitation of the study is the fact that the questionnaire was only assessed in one of the five study centers, limiting generalizability of the results, and that no data on gender or age of the students were available due to the anonymous data collection. Further limitations include the relatively short open text field answers assessed via questionnaire. In future studies, additional voluntary semi-structured interviews may provide more detailed insights into reasons for non-participation.

In conclusion, non-participation of C&A in research projects on mental health is a very important but largely underexplored research field. This study holds important insights for further research. Specifically, the reduction of stigma toward mental health, e.g., through targeted programs and interventions (18), holds promises for higher participation rates. Further, for school-based recruitment, teachers and school staff should be involved in the planning process as much as possible, e.g., by face-to-face meetings with school principals or at teacher conferences. In the present study, it was also offered to hold workshops on mental health related topics for students and school staff. Moreover, when targeting C&A, it is crucial to understand the key role of parents for the participation of minors (18) and to inform parents as early and as detailed as possible. Another way of reducing non-participation might be by offering incentives relevant to the targeted age group. In the present study, small incentives such as rulers or pencils with the project's logo were offered and students had the chance to win a voucher when having completed the questionnaire. Finally, offering a variety of ways to inform about the research project and to contact the research team, e.g., via different social media accounts, might be an easily accessible way for students to reach out if they have further questions about the study.

## Data availability statement

The raw data supporting the conclusions of this article will be made available by the authors, without undue reservation.

## Ethics statement

The studies involving humans were approved by Ethics Committee of the Medical Faculty, University of Leipzig. The studies were conducted in accordance with the local legislation and institutional requirements. Written informed consent for participation was not required from the participants or the participants' legal guardians/next of kin because the survey was completely anonymous and participation was voluntary.

## Author contributions

SBal: Writing—original draft, Writing—review & editing, Conceptualization, Data curation, Formal analysis, Investigation, Methodology. S-LK: Writing—original draft, Writing—review & editing, Conceptualization, Data curation, Formal analysis. EK: Writing—original draft, Writing—review & editing, Conceptualization, Data curation, Formal analysis, Investigation, Methodology. SM: Writing—original draft, Writing—review & editing, Formal analysis. SBau: Writing—original draft, Writing—review & editing. KB: Writing—review & editing. SD: Writing—review & editing. HE: Writing—review & editing. AH: Writing—review & editing. MK: Writing—review & editing. JK: Writing—review & editing. LL: Writing—review & editing. MM: Writing—review & editing. RT: Writing—review & editing. CR-K: Supervision, Writing—original draft, Writing—review & editing, Conceptualization, Data curation, Formal analysis, Funding acquisition, Investigation, Methodology, Project administration.

## The ProHEAD consortium

The ProHEAD consortium comprises six study sites in Germany. Site leaders are: Michael Kaess (University Hospital Heidelberg), Stephanie Bauer (University Hospital Heidelberg), Rainer Thomasius (University Medical Center Hamburg-Eppendorf), Christine Rummel-Kluge (University Leipzig), Heike Eschenbeck (University of Education Schwäbisch Gmünd), Hans-Joachim Salize (Medical Faculty Mannheim/Heidelberg University), and Katja Becker (Philipps-University Marburg). Further members of the consortium are: Sabrina Bonnet, Johannes Feldhege, Christina Gallinat, Stella Hammon, Julian Koenig, Sophia Lustig, Markus Moessner, Fikret Özer, Regina Richter, Johanna Stadler (all University Hospital Heidelberg), Steffen Luntz (Coordinating Center for Clinical Trials Heidelberg), Silke Diestelkamp, Anna-Lena Schulz (all University Medical Center Hamburg-Eppendorf), Sabrina Baldofski, Sarah-Lena Klemm, Elisabeth Kohls, Sophia Müller, Lina-Jolien Peter, Mandy Rogalla (all University Leipzig), Vera Gillé, Johanna Jade, Laya Lehner (all University of Education Schwäbisch Gmünd), Elke Voss (Medical Faculty Mannheim/Heidelberg University), Alisa Hiery, and Jennifer Krämer (all Philipps-University Marburg).
